# Characterization and biological activity of selenium nanoparticles biosynthesized by *Yarrowia lipolytica*

**DOI:** 10.1111/1751-7915.70013

**Published:** 2024-10-04

**Authors:** Elham Lashani, Hamid Moghimi, Raymond J. Turner, Mohammad Ali Amoozegar

**Affiliations:** ^1^ Extremophiles Laboratory, Department of Microbiology, School of Biology, College of Science University of Tehran Tehran Iran; ^2^ Department of Microbiology, School of Biology, College of Science University of Tehran Tehran Iran; ^3^ Department of Biological Sciences University of Calgary Calgary Alberta Canada

## Abstract

In this research, biogenic selenium nanoparticles were produced by the fungi *Yarrowia lipolytica*, and the biological activity of its nanoparticles was studied for the first time. The electron microscopy analyses showed the production of nanoparticles were intracellular and the resulting particles were extracted and characterized by XRD, zeta potential, FESEM, EDX, FTIR spectroscopy and DLS. These analyses showed amorphous spherical nanoparticles with an average size of 110 nm and a Zeta potential of −34.51 ± 2.41 mV. Signatures of lipids and proteins were present in the capping layer of biogenic selenium nanoparticles based on FTIR spectra. The antimicrobial properties of test strains showed that *Serratia marcescens*, *Klebsiella pneumonia*, *Escherichia coli*, *Pseudomonas aeruginosa* and *Bacillus subtilis* were inhibited at concentrations between 160 and 640 μg/mL, while the growth of *Candida albicans was hindered by* 80 μg/mL of biogenic selenium nanoparticles. At concentrations between 0.5 and 1.5 mg/mL of biogenic selenium nanoparticles inhibited up to 50% of biofilm formation of *Klebsiella pneumonia*, *Acinetobacter baumannii*, *Staphylococcus aureus* and *Pseudomonas aeruginosa*. Additionally, the concentration of 20–640 μg/mL of these bioSeNPs showed antioxidant activity. Evaluating the cytotoxicity of these nanoparticles on the HUVEC and HepG2 cell lines did not show any significant toxicity within MIC concentrations of SeNPs. This defines that *Y. lipolytica* synthesized SeNPs have strong potential to be exploited as antimicrobial agents against pathogens of WHO concern.

## Graphical abstract

The production of selenium nanoparticle by *Yarrowia lipolytica*, characterizing shape and size by different analyses and evaluation their biological activities.

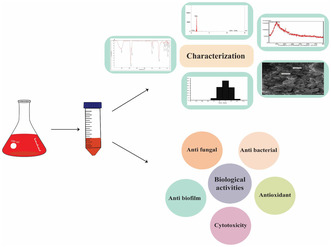

## INTRODUCTION

Particles that have dimensions below 100 nm in at least one or more dimensions are called nanoparticles (NP). Optimal surface‐to‐volume ratio and small size cause high surface performance and unique physical properties in nanoparticles (Klabunde, 1994; Yosri et al., 2021). These characteristics ultimately affect the catalytic activity and make NPs suitable candidates for use in many applications, such as medicine and biology (Bakhtiari‐Sardari et al., 2020; Baran, 2019), optical and electrical instruments, photovoltaic cells (Panahi‐Kalamuei et al., 2014), energy and gas storage (Ahmad et al., 2020) and catalysis (Rajagopal et al., 2021). Metal NPs (Gold et al., 2018) and selenium have become of increasing interest (for recent reviews see (Bisht et al., 2022)).

Selenium NPs are used in the biomedical field to treat diseases such as diabetes (Al‐Quraishy et al., 2015) and inflammatory issues (Malhotra et al., 2016). Also, many studies have been reported on the antioxidant (Forootanfar et al., 2014), antimicrobial (Yildirim et al., 2022), anticancer (Baran et al., 2023), anti‐leishmaniasis (Beheshti et al., 2013), and antibiofilm activity (Shakibaie et al., 2015) of Se NPs. Other applications of Se NPs are in dye degradation (Ahluwalia et al., 2016) and use for mercury bioremediation (Wang et al., 2019).

There are different physical and chemical methods to produce nanoparticles often requiring a lot of energy or producing hazardous waste (Jamkhande et al., 2019). An alternative is the use of biological systems, particularly microorganisms to produce nanoparticles in a green, eco‐friendly, and cost‐effective approach. Many studies have investigated the production of selenium nanoparticles using a variety of bacterial species (Baggio et al., 2021; Borghese et al., 2014; Bulgarini et al., 2021; Lampis et al., 2017; Piacenza et al., 2018; Presentato et al., 2016, 2018; Zonaro et al., 2015) while the study of yeasts for Se nanoparticle biosynthesis has been less investigated and limited to a few genera such as *Saccharomyces*, *Magnusiomyces*, and *Rhodotorula* (Ashengroph & Tozandehjani, 2022; Ruocco et al., 2014; Salem, 2022). For some time, *Saccharomyces* species have been used to produce selenium‐rich yeasts, which are used as food supplements for humans and livestock grown in Se poor regions (Kieliszek et al., 2015). Determining which approach is superior, bacteria or fungi, to produce metal NPs is presently under debate and likely rests with researchers bias and what the targeted application will be, but it is critical to consider what the NPs will carry over from their biogenic production host (such as immune responsive biomolecules from bacteria).

An important observation supporting biogenic production over chemical/physical production of NPs is that for reasons not fully appreciated yet, the biogenic NPs are often more antimicrobial than the chemically synthesized. In the case of SeNPs, the biogenic SeNPs were more antimicrobial compared with chemogenic against a variety of bacterial of concern including, *P. aeruginosa*, *S. aureus*, *E. coli* and *K. pneumoniae* (Piacenza et al., 2017), and these SeNPs have the ability to inhibit the development of biofilms.

Production of Se NPs in *Rhodotorula mucilaginosa* R‐8441 ranged in size from 83 to 478 nm proportional to added selenite concentration which also changed the shape of the SeNPs from spherical to nanorods (Ashengroph & Tozandehjani, 2022). In another study, the cell‐free extract of *Magnusiomyces ingens* LH‐F1 was used by Lian et al. (2019) to generate, spherical and quasi‐spherical SeNPs with an average diameter of 87.82 ± 2.71 nm and hexagonal crystalline structure with a low molecular weight protein as a stabilizer. In a study performed using *Saccharomyces cerevisiae*, intracellular SeNPs were produced and were extracted with a diameter between 75 and 709 nm with a polydispersity index 0.189 to 0.989, and zeta potential ranging from −7.06 to −10.3 mV depending on starting conditions (Faramarzi et al., 2020). Spherical selenium nanoparticles were produced by *S. cerevisiae* with a size range of 4–51 nm and were found to be associated with a protein cap. The antimicrobial activity of these NPs against food‐borne pathogens was investigated and bacterial species such as *Escherichia coli*, and *Staphylococcus aureus* were inhibited in concentrations of 62.5, and 125 μg/mL, respectively. However, higher amounts of the Se NPs were needed to inhibit the fungal genus *Aspergillus* (250 and 500 μg/mL for *Aspergillus fumigatus* and *Aspergillus niger*, respectively) (Salem, 2022).

The fungal yeast *Yarrowia lipolytica* is known as a grass microorganism with the ability to produce a high amount of biomass with the ability to degrade hydrocarbons and produce fatty acids (Hassanshahian et al., 2012). Also, *Yarrowia* can be used as a food supplement for providing vitamins, trace elements, high amounts of fatty acids, exogenous amino acids and proteins (for further information see Jach and Malm (2022)) (Jach et al., 2021; Yang et al., 2022). Hence, using *Y. lipolytica* for the biosynthesis of Se NPs can be important in terms of ease and safety of culturing as well as the useful biomolecules that could become associated with the NP cap. Since this organism is proven to be biocompatible there is less risk of toxins transferred on the NP in biomedical applications. However, only one study has been conducted on using selenium‐enriched *Yarrowia* as a food for improving the health condition of the plankton *Artemia salina* (Hamza et al., 2017).

The aim of this study was the synthesis of bioSeNPs by the fungi strain *Y. lipolytica* and then study their characteristics and particularly their biological activities. Because of the emergence of multidrug‐resistant microorganisms, finding new antimicrobial compounds is necessary. Hence, bioSeNPs produced by *Y. lipolytica* were applied against Gram‐negative and ‐positive bacteria to evaluate their antimicrobial efficacy. Also, the ability of these nanoparticles to scavenge radicals was investigated to evaluate whether they could prevent oxidative stress damage if applied to a wound situation. Preventing biofilm formation by bacteria was also investigated, as well as the toxicity of these nanoparticles against the tissue culture cell lines was studied. This is the first report of using the yeast *Y. lipolytica* in the study of bioSeNPs; we consider the potential of this organism for biogenic production superior to others due to its non‐pathogenic characteristics, biocompatibility as a probiotic and robust growth under a variety of conditions.

## RESULT AND DISCUSSION

### BioSeNPs biosynthesis by *Y. Lipolytica*

**Figure 1:**
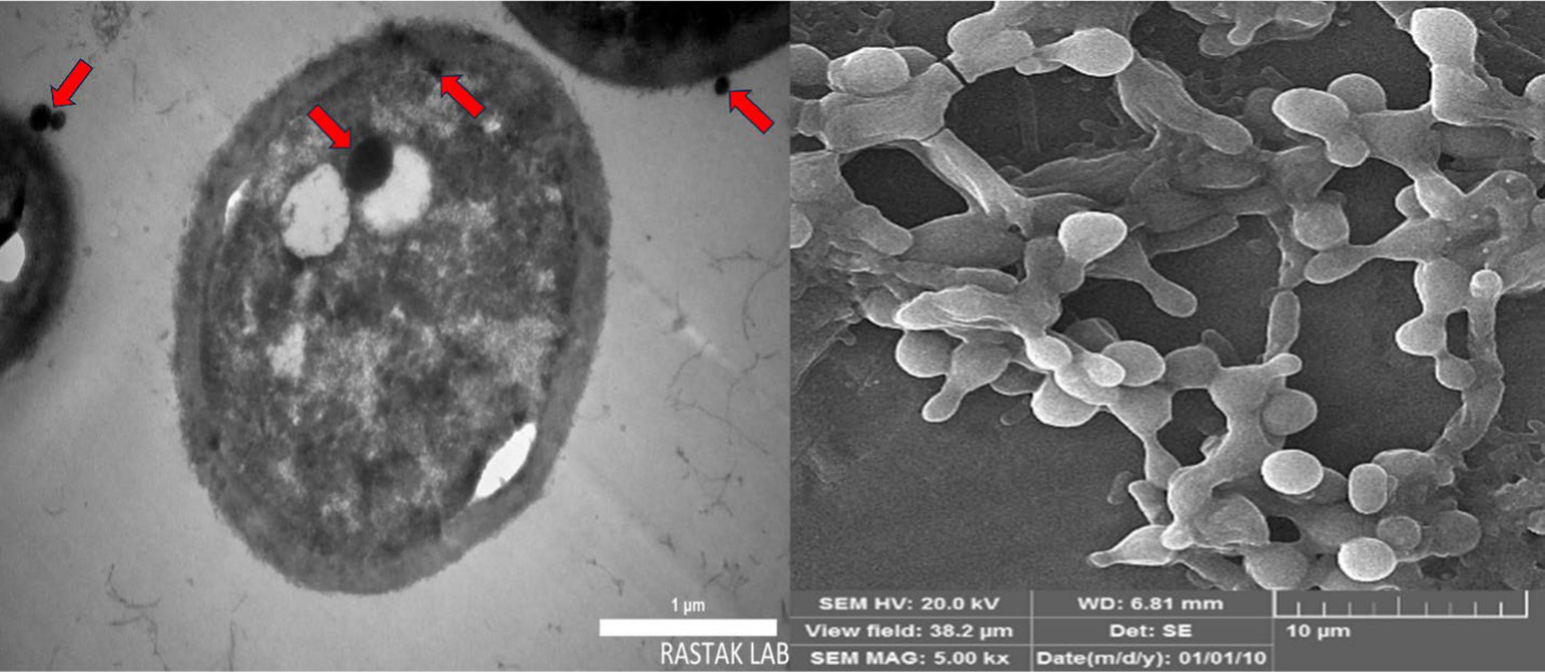
Transmission electron microscopy (left) and scanning electron microscopy (right) micrographs of *Y. lipolytica* in the presence of 1 mM selenite (black points show selenium nanoparticles).

The yeast *Yarrowia lipolytica* was cultured on a GYP broth medium containing selenite and the appearance of a light red colour showed bioSeNPs production. Also, electron microscopy was applied to detect the location of selenium nanoparticle synthesis (Figure 1). The thin section of *Y. lipolytica* revealed intracellular accumulations of Se(0). Some of these nanoparticles were associated with the cell membranes. These data indicated that Se nanosphere formation and selenite reduction occurred in different parts of the cells indicating that several nucleation points can exist inside the *Y. lipolytica* cell for selenite bioreduction.

The mechanism of selenium nanoparticle biosynthesis in *Y. lipolytica* has not been investigated yet. Other yeasts, including *S. cerevisiae* and *Candida* species showed the role of different transporters, such as sulphate permease (Sul1p and Sul2p), phosphate transporters (Pho87p, Pho90p and Pho91p) and Jen1p monocarboxylic conveyor in transporting selenite into the cell. After entry, it is expected that selenite will be reduced to Se(0) by glutathione and other thiol containing proteins such as thioredoxin (Kieliszek et al., 2015; Lazard et al., 2010; McDermott et al., 2010).

### Characterization of the biogenically produced SeNPs

**Figure 2:**
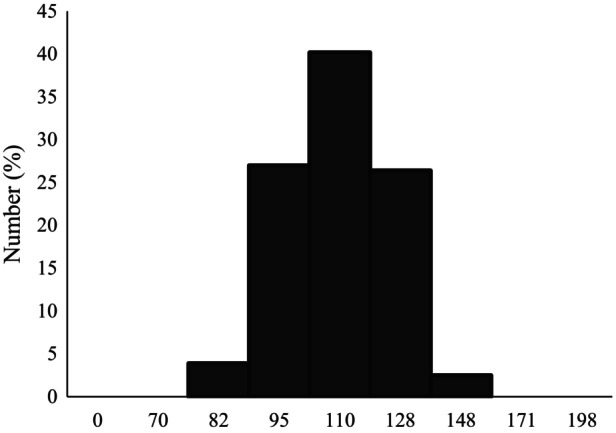
Particle size distribution of selenium nanoparticles by dynamic light scattering (DLS) analysis. Representative data from three biological replicates (Standard deviation is 14.03).

**Figure 3:**
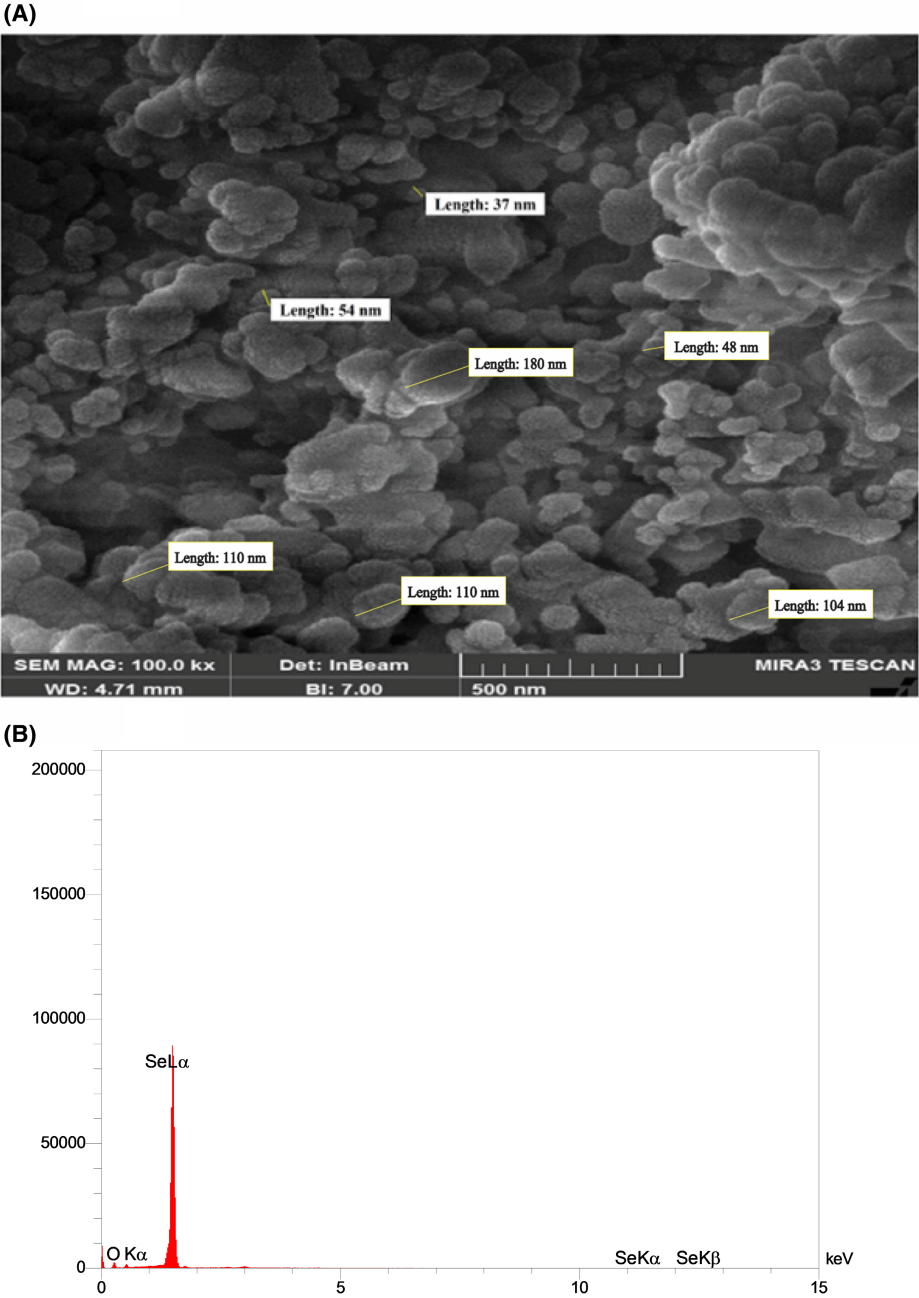
Scanning electron microscopy images (A) showing the size and shape of purified biogenic selenium nanoparticles and energy dispersive spectroscopy (B) of biogenic selenium nanoparticles showing the signature peaks of selenium, Lα, Kα, and Kβ.

After collecting the biomass and purifying the nanoparticles, various methods were applied to determine the properties of the produced nanoparticles. The average size of nanoparticles was obtained by dynamic light scattering (DLS) and was 110 nm with a polydispersity index (PDI) of 1 (Figure 2). The Zeta potential analyser measured the surface charge of bioSeNPs, and the value of average zeta potential was determined at −34.51 ± 2.41 mV. Nanoparticles with zeta potential between ±20–30 mV have a moderately stable nature, and higher values show more stability, thus *Y. lipolytica* produces very stable uniform SeNPs. FESEM showed many spherical nanoparticles with variable sizes and amorphous nature (Figure 3A). Also, EDX analysis confirmed the presence of bioSeNPs (Figure 3B) which exhibited a strong absorption peak at 1.37 keV.

Nanoparticles have different biological properties and activities due to various functional groups provided by the biomolecules that become associated with their surface and are referred to as the capping layer. FTIR analysis was applied for identifying these functional groups originating from yeast biomolecules that are involved in the biotransformation of selenite into bioSeNPs. The capping layer of biogenic nanoparticles confers them distinct important features, such as controlling the size of nanoparticles by preventing their aggregation and agglomeration, enhancing stability that enables their long‐term storage. Also, the capping layer plays a critical role in defining the biological activities of nanoparticles by interacting with the biomolecules presented on the surface of target cells and making them a suitable tool for biomedical properties, such as drug delivery, anticancer and antimicrobial activities (Bulgarini et al., 2021; Jain et al., 2015; Piacenza et al., 2023). In an interesting study, Cremonini et al. (2018) investigated the role of the cap layer of bioSeNPs produced by *Stenotrophomonas maltophilia* SeITE02 and their findings indicated that by detaching the biochemical cap layer, which is made of proteins and carbohydrates, the stability of the nanoparticles significantly decreased. Also, their loss has a negative effect on the antibiofilm and antimicrobial properties of bioSeNPs (Cremonini et al., 2018).

**Figure 4:**
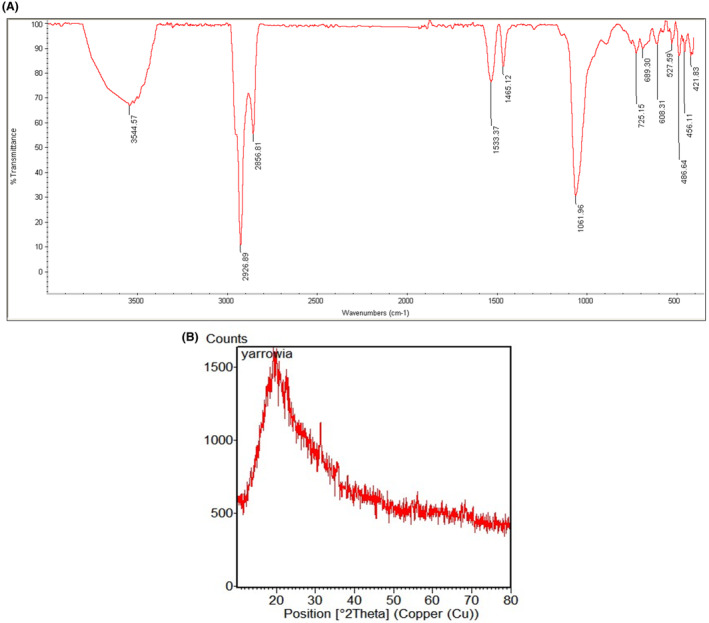
(A) Fourier transform infrared (FTIR) and (B) X‐ray diffraction (XRD) spectra of the biogenic selenium nanoparticles.

Based on the FTIR spectra (Figure 4A), the broad peak of 3544 cm^−1^ and the peaks of 2926 and 2856 cm^−1^ refer to OH or NH groups, CH_2_ asymmetric stretching of long chain aliphatic compounds, and CH_2_ symmetric stretching, respectively. Also, the peaks of 1533 and 1465 cm^−1^ showed amide and CH_2_/CH_3_ bending vibrations in lipids and proteins. The peak of 1061 cm^−1^ indicated carboxylic group of fatty acids, and proteins and phosphate ions. Based on our findings and similar to SeNPs biosynthesized by fungus *Mariannaea* sp. HJ (Zhang et al., 2019), and *S. maltophilia* SeITE02 (Lampis et al., 2017), *Bacillus amyloliquefaciens* SRB04 (Ashengroph & Hosseini, 2021), we conclude that lipids and proteins are also present in the capping layer of bioSeNPs produced by *Y. lipolytica*.

BioSeNPs with a capping layer of protein are also biosynthesized by different microorganisms, such as *Bacillus licheniformis* (Khiralla & El‐Deeb, 2015), *Streptomyces* sp. (M10A65) (Ramya et al., 2020), *Paenibacillus motobuensis* LY5201 (Long et al., 2023), *Acinetobacter* sp. SW30 (Wadhwani et al., 2017), *Streptomyces bikiniensis* strain Ess_amA‐1 (Ahmad et al., 2015), *Penicillium expansum* ATTC 36200 (Hashem et al., 2021), *Bacillus licheniformis* (Khiralla & El‐Deeb, 2015), *Bacillus cereus* (Kora, 2018), *Pseudomonas aeruginosa* ATCC 27853 (Kora & Rastogi, 2016), *Rhodococcus aetherivorans* BCP1 (Ramya et al., 2015) *Streptomyces olivaceous* S91 (Elshaer & Shaaban, 2021), *Saccharomyces cerevisiae* (Salem, 2022) and *Bacillus* sp. B2 (Bharathi et al., 2020).

Polysaccharide is the other biomolecule present in the capping layer of bioSeNPs produced by *Rhizopus oryzae* (Abu‐Elghait et al., 2021) and *Spirulina* (Yang et al., 2012). Also, different microorganisms, such as *Azospirillum brasilense* Sp7, *Proteus* sp. YS02 (Wang et al., 2022), *Azospirillum thiophilum* (Tugarova et al., 2018), *Rhodotorula* sp. strain MZ312359 (El‐Fakharany et al., 2023), *Achromobacter* sp. R2A, *Stenotrophomonas maltophilia* SeITE02, *Bacillus mycoides* SeITE01, *Lysinibacillus* sp. R1E (low carbohydrate content) and *Ensifer* sp. R2D (Bulgarini et al., 2021) produced nanoparticles with a capping layer composed of lipids, protein and polysaccharides. Protein and polysaccharides are present in the capping layer of *Lactobacillus casei* 393 (Xu et al., 2018).

The pattern of XRD analysis showed that bioSeNPs produced by *Y. lipolytica* were noisy and without any sharp Bragg reflection, which indicated that they have an amorphous character (Figure 4B). These results are similar to the earlier XRD analysis performed on SeNPs biosynthesized with *Bacillus mycoides* SeITE01 (Lampis et al., 2014), *Pseudomonas aeruginosa* ATCC 27853 (Kora & Rastogi, 2016) and *Lactobacillus casei* ATCC 393 (Spyridopoulou et al., 2021). In Table 1, a summary review of characterization results of microbial‐produced selenium nanoparticles is presented for comparison.

**Table 1:** Summary of biogenic selenium nanoparticle characterization.

Source of biosynthesis	Size	Shape	Nature	Applications	References
*Acinetobacter* sp. SW30	79, and 78 nm	Polygonal, nanorods, nanosphere	Amorphous (nanospheres) and crystallite (nanorods)	Anticancer	Wadhwani et al. (2017)
*Streptomyces bikiniensis* strain Ess_amA‐1	17 nm	Nanorods	‐	Anticancer	Ahmad et al. (2015)
*Spirulina*	20–50	Spherical	‐	Anticancer	Yang et al. (2012)
*Penicillium expansum* ATTC 36200	4–12.7 nm	Spherical	Crystallite	Antibacterial, antifungal, and antioxidant	Hashem et al. (2021)
*Bacillus licheniformis*	10–50 nm	Spherical	Face‐centred cubic	Antibiofilm, antimicrobial	Khiralla and El‐Deeb (2015)
*Lactobacillus casei* 393	50–80 nm	Spherical	‐	Anticancer and antioxidant	Xu et al. (2018)
*Ralstonia eutropha*	40–120 nm	Spherical	Crystalline hexagonal and actinomorphic trigonal nanorods	Antimicrobial	Srivastava and Mukhopadhyay (2015)
*Idiomarina* sp. PR58‐8	150–350 nm	Spherical	Hexagonal	Anticancer	Srivastava and Kowshik (2016)
*Citrobacter fruendii* strain KP6	45–70 nm	Spherical	Crystalline	‐	Samant et al. (2018)
*Anabaena variabilis* NCCU‐441	10.8 nm	Spherical	Amorphous	Antioxidant and antimicrobial	Afzal et al. (2021)
*Vibrio natriegens*	100–400	Spherical	‐	‐	Fernández‐Llamosas et al. (2016)
*Bacillus cereus*	50–150 nm	Spherical	Amorphous	‐	Kora (2018)
*Enterococcus faecalis*	29–195 nm	Spherical	‐	Antibacterial	Shoeibi & Mashreghi (2017)
*Bacillus pumilus* sp. BAB‐3706	10–80 nm	Spherical	Crystalline	Biosensor	Prasad et al. (2015)
*Pseudomonas aeruginosa* ATCC 27853	47–165 nm	Spherical	Amorphous	‐	Kora and Rastogi (2016)
*Rhodococcus aetherivorans* BCP1	10–250 nm	Spherical	Crystalline	Antioxidant, antibiofilm, wound healing, antiviral, and anticancer	Ramya et al. (2015)

### Antimicrobial activity of biogenic selenium nanoparticles

The use of biogenically produced nanoparticles is a green approach to producing new antimicrobials and is a good way to deal with microbial resistance to antibiotics, which is considered a severe problem in clinical microbiological studies (Arora et al., 2024). For this purpose, the obtained bioSeNPs were tested for their antimicrobial activity against Gram‐positive (*B. subtilis*) and Gram‐negative bacteria (*K. pneumonia*, *E. coli*, *P. aeruginosa* and *S. marcescens*) by using the microdilution method (Balouiri et al., 2016). The results are shown in Table 2. Among the bacterial species tested, *S. marcescens* was the most susceptible strain with an MIC of 160 μg/mL, while the growth of other bacterial strains and the yeast *C. albicans* was inhibited in 640 and 80 μg/mL of bioSeNPs, respectively.

**TABLE 2:** Minimal inhibitory concentrations (MIC) of selenium nanoparticles against Gram‐negative and Gram‐positive bacteria and the fungi *C. albicans*.

Domain	Microorganisms	MIC (μg/mL)
Bacteria	*B. subtilis*	640
	*K. pneumonia*	640
	*E. coli*	640
	*P. aeruginosa*	640
	*S. marcescens*	160
Fungi	*Candida albicans*	80

*Note*: Results from three replicates.

It is not clear how SeNPs lead to bacterial cellular toxicity, but it is known there is membrane fluidity disruption upon interaction with eukaryotic model lipid membrane (Piacenza et al., 2023). There is the possibility that upon interaction with a cell the NPs decompose oxidizing the Se back to Selenite. Selenite has antibacterial effects by oxidizing thiols resulting in cellular dysfunctions. SeNPs regardless of particle size and morphology typically lead to oxidative stress although the mechanism for this is not clear. However, the toxicity of nanoparticles depends on what species are used to produce them (Menon et al., 2019). Zhang et al. (2021) investigated the antimicrobial activity of bioSeNPs produced by *Providencia* sp. against *S. aureus*, *Bacillus cereus*, *B. subtilis*, *P. aeruginosa*, *E. coli* and *Vibrio parahemolyticus*. The microbial synthesized SeNPs with a size of 120 nm significantly inhibited the test strains, mainly Gram‐negative bacteria with a concentration of about 500 μg/L. Observations of these nanoparticles on the bacteria were oxidative damage, changing membrane permeability and disrupting the bacterial cell wall (Zhang et al., 2021).

Another study by Khiralla and El‐Deeb (2015) showed the antibacterial properties of bioSeNPs against food‐borne bacteria, including *S. aureus*, *E. faecalis* and *B. cereus*. A minimum 90% inhibitory concentration (MIC_90_) of these nanoparticles with 10–50 nm size was reported at 25 μg/mL (Khiralla & El‐Deeb, 2015). The antibacterial properties of nanoparticles depend on their size, and smaller nanoparticles show higher antimicrobial activity. Smaller nanoparticles have a higher surface area‐to‐volume ratio, thus giving a bulk effect that they can adsorb more molecules compared with larger nanoparticles. Furthermore, ROS generation of smaller nanoparticles is higher and makes them a suitable candidate for antimicrobial applications (Zhang et al., 2021).

*Candida albicans* was the pathogenic yeast that was selected for our study because of causing infection in the urinary tract, GI tract and oral cavity (Menon et al., 2019). The results in our study showed that a low amount of our bioSeNPs can inhibit *C. albicans*. A study using *Lactobacillus* sp. was used for bioSeNPs biosynthesis, that gave results similar to our data and MIC and MFC results showed that *C. albicans* was inhibited by Se NPs with concentrations of 55 and 100 μg/mL, respectively (El‐Saadony et al., 2021).

San Keskin et al. (2020) used microbially synthesized SeNPs (spherical shape, 130 nm) against *E. coli* ATCC 125922 and *S. aureus* ATCC 29213 with a concentration of 1 mg/mL, and their results showed that SeNPs could prevent these two bacteria with the inhibition rate of 80.8 ± 0.12%, and 87.7 ± 0.23%, respectively (San Keskin et al., 2020). Different concentrations of SeNPs (40–200 μg/mL) were applied against various bacteria, including *S. aureus*, *B. subtilis*, *Acinetobacter* sp., *K. pneumonia*, *P. aeruginosa* and *E. coli*. The best results were obtained when they used 200 μg/mL of SeNPs illustrating the antibacterial activity of the NPs is dose‐dependent (Ramya et al., 2020). Abu‐Elghait et al. (2021) biosynthesized SeNPs using *Rhizopus oryzae*. They investigated the antimicrobial effect of these nanoparticles against *S. aureus*, *B. subtilis*, *E. coli*, *P. aeruginosa*, *S. typhimurium* and *C. albicans*. All pathogens were inhibited at bioSeNPs concentrations less than 3000 ppm (Abu‐Elghait et al., 2021).

Filipović et al. (2021) produced SeNPs by different chemical reduction approaches using chitosan + ascorbic acid, BSA + ascorbic acid and with glucose as stabilizers and reducing agents. Their results showed that all bacterial strains such as *P. aeruginosa*, *S. aureus*, *Kocuria rhizophila*, *B. subtilis*, *E. coli*, *E. faecalis*, *K. pneumoniae* and *Salmonella abony* were inhibited with the concentration of 100–400 μg/mL of the chemically synthesized SeNPs. In contrast, only a range of 25–72 μg/mL of all differently produced nanoparticles were needed for growth inhibition of *C. albicans* (Filipović et al., 2021).

The precise mechanism(s) underlying the antimicrobial action of metal‐based antimicrobial nanoparticles is confused in the literature which may be a result of the diversity of nanomaterial core metals, structures, capping material and stability. These issues are further complicated by differences between physical‐chemically produced NPs vs biogenic NPs which is particularly clear for Se NPs (Piacenza et al., 2017). Regardless, proposed mechanisms include the deterioration of cell membrane integrity, increased generation of reactive oxygen species (ROS) and DNA damage. These effects collectively lead to the suppression of cell growth and eventually cell death (Huang et al., 2016).

### Inhibition of biofilm formation by bioSeNPs

Biofilm formation by pathogenic bacterial strains causes several problems and increases bacterial resistance to antimicrobial compounds such as antibiotics. In this regard, the inhibitory effects of bioSeNPs against biofilm attachment by *P. aeruginosa*, *S. aureus*, *K. pneumonia* and *A. baumannii* were investigated by using microplate experiment (Allkja et al., 2020). The results showed that 1 mg/mL bioSeNPs could inhibit 51.37% of *K. pneumonia* biofilm. Biofilm of *S. aureus* was the most susceptible to bioSeNPs, and as shown in Table 3, 0.5 mg/mL of bioSeNPs prevented almost 40% of its formation. In a similar study by Shakibaie et al. (2015).

**Table 3:** Inhibition of biofilm formation of different bacteria by the bioSeNPS.

Strain	Concentration (mg/ml)	Percentage of inhibition (%) ± S.D
*A. baumannii*	1.5	42.5 ± 6.5
*P. aeruginosa*	1	40 ± 3.6
*S. aureus*	0.5	38 ± 2.5
*K. pneumonia*	1	51.3 ± 6.5

*Note*: Results from three replicates.

The effect of SeNPs on the biofilm formation of different bacterial strains was qualitatively investigated by Khiralla et al. (2015). In their study, different concentrations of SeNPs were applied against *Bacillus cereus*, *Enterococcus faecalis*, *S. aureus*, *E. coli* O157:H7, *Salmonella typhimurium* and *Salmonella enteritidis*. Their results showed that all biofilm formation of strains is inhibited in a concentration less than 20 μg/mL except *B. cereus* (Khiralla & El‐Deeb, 2015).

In another study, the ability to prevent biofilm formation of biogenic SeNPs synthesized by *Bacillus mycoides* and *Stenotrophomonas maltophilia* are compared with their chemical counterpart. This study was conducted on seven different strains of *P. aeruginosa* (clinical and reference strains) and 500 μg/mL of SeNPs produced by *B. mycoides* and *S. maltophilia* prevented biofilm formation in the range of 64%–96%, while this range for chemical SeNPs was between 30% and 97% (Cremonini et al., 2016).

Sonkusre and Cameotra (2015) reported that by increasing the concentration of SeNPs from 0.1 to 0.5 mg/mL, the ability of SeNPs to hinder the biofilm formation of *S. aureus* was significantly improved. They also proposed that this remarkable property of SeNPs could inhibit biofilm production in medical‐related devices (Sonkusre & Cameotra, 2015). In a study by Abu‐Elghait et al. (2021), 150 ppm of SeNPs had an inhibitory effect on the biofilm formation of *S. aureus* and *P. aeruginosa* by 90.1 ± 3.6% and 40.1 ± 1.7%, respectively (Abu‐Elghait et al., 2021). Different concentrations of SeNPs (0.5, 1, 1.5 and 2 mg/μl) were applied to inhibit the formation of *P. aeruginosa* biofilm. The results indicated that increasing concentration effectively impacts biofilm inhibition. The best result was obtained in 2 mg/μl of SeNPs, and biofilm thickness decreased by 50% (San Keskin et al., 2020).

### Cytotoxicity of SeNPs on HepG2 and HUVEC cell line by the MTT assay

**Figure 5:**
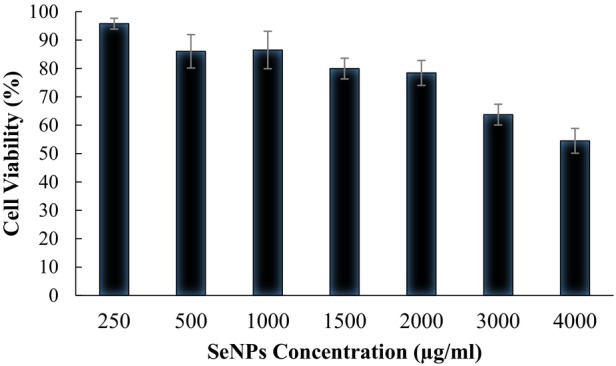
Cytotoxic effect of bioSeNPs on HepG2 cells (*N* = 3, and error bars showed standard deviation).

Treatment with different concentrations of SeNPs was conducted on the HUVEC and HepG2 cell line to measure the cytotoxicity of nanoparticles and viability of cells after treatment. As shown in Figure 5, SeNPs produced by *Y. lipolytica* in concentration near our determined MIC did not show any cytotoxic effect on both cell lines. Although bioSeNPs produced by *Y. lipolytica* had no toxic effects on HUVEC cells through various concentrations (0.5–4 mg/mL), the apparent inhibitory concentration at 50% of maximum (IC50) to the HepG2 cell line was around 4 mg/mL. Guo et al. (2017) investigated the effect of cSeNPs (chemically synthesized selenium nanoparticles) and cSeNPs decorated with curcumin on HepG2 cell culture. Although they did not mention the SeNPs concentration used for this experiment, no specific effect was observed compared with untreated cells reflecting the influence of the cap biomolecules. Furthermore, their result showed that the decoration of SeNPs with curcumin increased the toxicity of nanoparticles (Guo et al., 2017). Xu et al. (2018) produced bioSeNPs by *L. casei* ATCC 393 and investigated the effect of these nanoparticles on HepG2 cell culture. Their results showed cytotoxicity of bioSeNPs at the concentration of 4, 8 and 16 μg/mL (Xu et al., 2018). Also, the result of a study conducted by Ahmad et al. (2015) showed LD_50_ of 75.96 for selenium nanorods (Ahmad et al., 2015).

### Antioxidant effect of SeNPs

**Figure 6:**
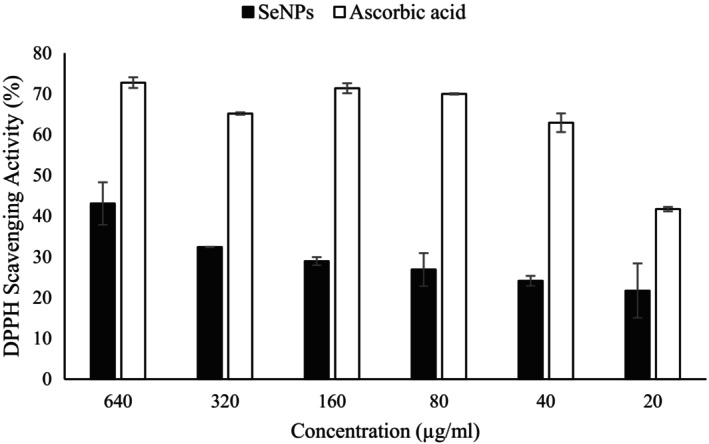
DPPH Scavenging Activity of SeNPs compared with ascorbic acid (*N* = 3, and error bars showed standard deviation).

The antioxidant activity of SeNPs was investigated by the DPPH assay as a rapid and precise method that helps measure the electron scavenging property of the SeNPs. When a compound has reductant antioxidant activity, DPPH colour changes from purple to yellow and is analysed by absorbance at 517 nm (Menon et al., 2019). The antioxidant activity of the bioSeNPs in comparison with ascorbic acid as a known oxidant scavenger agent is shown in Figure 6. Our results showed that by increasing the concentration of bioSeNPs from 20 to 640 μg/mL, the antioxidant activity of these nanoparticles increases from 21% to 43%.

Chemically synthesized SeNPs were produced by Dumore and Mukhopadhyay (2020) using gallic acid as a reducing agent. In their study, the antioxidant activity of spherical‐shaped SeNPs with a size of 98 nm and concentration of 207.6 μg/mL was investigated. Their results showed a 73% scavenging activity percentage at neutral pH (Dumore & Mukhopadhyay, 2020). Forootanfar et al. (2014) produced SeNPs (spherical with the size of 80–220 nm) using *Bacillus* sp. MSh‐1. Antioxidant activity of 200 μg/mL of these nanoparticles was reported at 23.1 ± 3.4% (Forootanfar et al., 2014). In another study using a cell‐free extract of *Geobacillus* as a stabilizing and reducing agent, SeNPs show higher scavenging activity (60 ± 4%) compared with sodium selenite (22.4 ± 4%) (Kumar et al., 2020).

In a study by Ramya et al. (2015), 100–1000 μg/mL of Actinobacteria synthesized SeNPs was applied to evaluate their antioxidant activity. Their results showed that with increasing concentration of SeNPs, more antioxidant activity was observed. The lowest scavenging activity (about 30%) was obtained when 100 μg/mL of SeNPs were used, and in contrast, a concentration of 1000 μg/mL of SeNPs showed the highest activity (about 90%) (Ramya et al., 2020). Antioxidant activity of biologically synthesized SeNPs using *Anabaena variabilis* NCCU‐441 compared to chemically synthesized ones were investigated, and the results showed higher scavenging activity of biogenic SeNPs (IC_50_ 83.89 ± 2.11 μg/mL in comparison with 174.79 ± 0.29 μg/mL for chemically synthesized SeNPs) (Afzal et al., 2021). In a similar study, the antioxidant activity of SeNPs (200 μg/mL) was investigated by Akçay and Avcı (2020), and their result showed that 37.6 ± 2.5% scavenging activity and IC50 of 322.8 μg/mL. Also, their finding showed that the increasing concentration of SeNPs led to an improvement in the antioxidant activity of these nanoparticles (Akçay & Avcı, 2020). In addition to concentration, the size of nanoparticles is another critical factor that influences antioxidant activity. A study by Faramarzi et al. (2020) on SeNP production by *S. cerevisiae* confirmed this as their results indicated that smaller SeNPs had better free radical capturing activity. Also, the initial concentration of selenite for the production of SeNPs is correlated with the antioxidant activity of obtained nanoparticles and by increasing 5‐fold in using initial selenium salt (from 5 to 25 μg) for SeNPs biosynthesis, the antioxidant activity decreases from 48.5% to 21.7% (Faramarzi et al., 2020).

## EXPERIMENTAL PROCEDURES

### Chemicals and instruments

Chemicals and instruments used in this study, included Sodium selenite (sigma), 1‐octanol (Chem‐Lab), nutrient broth (Himedia), glucose (Himedia), yeast extract (Himedia), SEM (VEGA3 TESCAN, *Czech Republic*), XRD (PHILIPS_PW1730, Netherlands), FESEM (TESCAN MIRA3, *Czech Republic*), FTIR (Thermo‐AVATAR, USA), DLS (Malvern Instruments Ltd) and Zeta plus zeta potential analyser (Brookhaven Instruments, Sweden).

### Pathogenic bacteria and yeast using in this study

*Pseudomonas aeruginosa* (ATCC9027), *Acinetobacter baumannii* (ATCC BAA‐747), *Escherichia coli* (ATCC 8739), *Klebsiella pneumoniae* (ATCC 13883), *Serratia marcescens* (ATCC 13880), *Staphylococcus aureus* (ATCC25923), *Bacillus subtilis* (ATCC6633) and *Candida albicans* (ATCC10231) were sourced from the ATCC collection and housed in the microbial collection of Environmental Biotechnology Lab (EBL) of University of Tehran.

### Microbial culture condition

*Yarrowia lipolytica* (ATCC 18942) was obtained from the Fungal Collection of Environmental Biotechnology Lab (EBL) of the University of Tehran. This strain was cultured on GYP agar medium containing (g/L, pH 7.0), 20, glucose; 10, yeast extract; 5, peptone; 20, agar; and 1000 mL, distilled water and incubated at 30°C for 2 days. The ability of reducing selenite was investigated qualitatively by culturing on GYP agar medium containing 2.5 mM sodium selenite. Also, this strain was cultured on GYP agar medium without selenite for investigating pigmentation (Lashani et al., 2023).

### Nanoparticle biosynthesis and extraction and electron microscopy

For nanoparticle biosynthesis, *Y. lipolytica* was inoculated in a GYP broth medium (pH 7.0) containing selenite (2.5 mM) and incubated at 160 rpm and 30°C for 2 days.

For electron microscopy analyses, *Y. lipolytica* was cultured in a GYP medium containing selenite (1 mM). After 3 days of incubation at 30°C, centrifugation of culture medium was carried out at 6000 × *g* (15 min), and the collected biomass was washed with phosphate buffer for two times and was divided in two portions. The glutaraldehyde (2% v/v) was prepared in 1.5 mM phosphate buffer and added to one of the portions for fixing the biomass, and the sample was incubated for 24 h. After LR White resin embedding, thin sections were taken and their analysis was carried out by Philips EM208S (Netherlands), which was operated at 100 kV. After adding glutaraldehyde to the other portion, dehydration of biomass was conducted by increasing the concentration of ethanol (25, 50, 75 and 100%, respectively), and the samples were analysed by SEM at 20 kV after coating with gold (Hosseini, Lashani, & Moghimi, 2023; Lashani et al., 2023).

Biomass of *Y. lipolytica* was collected, and nanoparticle extraction was conducted by the method of Bulgarini et al. (2021) with a few modifications. Briefly, cultures were centrifuged for 10 min at 12,000 × *g* and twice washed with 0.9% NaCl. Then, cells were collected, frozen and lysed by adding liquid nitrogen, and they were sonicated for 3.5 min. After centrifugation for 20 min at 4300 × *g* and 4°C, the debris was removed. 2 mL of 1‐octanol was added to the supernatant containing SeNPs for every 5 mL lysate aliquot. After agitating the mixture and centrifuging it for 5 min at 480 × *g*, it was allowed to stay overnight at 4°C. SeNPs were separated from the aqueous phase by centrifuging at 18,000 × *g* for 20 min. Obtained nanoparticles were kept in Tris–HCl buffer (pH 7.4) at 4°C for further analyses (Bulgarini et al., 2021; Hosseini, Lashani, & Moghimi, 2023).

### Biogenic selenium nanoparticle characterization

DLS analysis was applied to determine the distribution and size of selenium nanoparticles. The structure of the selenium nanoparticle was investigated by XRD (40 kV, 30 mA) technique. This technique gives us information about the crystalline structure of nanoparticles. When a nanoparticle has an amorphous structure, no diffraction peak is observed by XRD analysis and does not help identify nanoparticle bulk size and structure. FESEM was applied to evaluate nanoparticle morphology and topography. Furthermore, this technique gives information about nanoparticle size. EDX is other technique that provides information about the elemental composition of metal nanoparticles. Element is characterized by a specific set of peaks in this technique. Selenium nanoparticles were fixed by glutaraldehyde (2% v/v) and were dehydrated subsequently by different concentrations of ethanol (25, 50, 75 and 100% v/v, respectively), and analysis was conducted by FESEM (Hosseini, Hadian, et al., 2023).

Another technique for characterizing nanoparticles is FTIR spectroscopy applied to analyse functional nanoparticle groups. Nanoparticle involvement during reduction is another piece of information provided by FTIR. Identifying these functional groups is essential for the determination of the route of nanoparticle biosynthesis. The measurements were performed in a range of 400–4000 cm^−1^. The surface charge of the bioSeNPs was determined at room temperature using a Zeta plus zeta potential analyser. Also, this instrument is used to measure these nanoparticles' average size (Hosseini, Hadian, et al., 2023; Hosseini, Lashani, & Moghimi, 2023).

### Antimicrobial effect of biogenic selenium nanoparticles

The antibacterial activity of bioSeNPs was studied by microdilution method against, *S. marcescens*, *E. coli*, *P. aeruginosa*, *B. subtilis and K. pneumonia*. In this method, bacterial species were cultured in a nutrient broth medium and incubated overnight 160 rpm at 37°C. A concentration equivalent to 0.5 Mac Farland of each strain was prepared and inoculated to 96‐well plates containing nutrient broth and serial dilution of selenium nanoparticles (10–1280 μg/mL). Different concentrations of bioSeNPs were used as a blank control (Barani et al., 2021; Eramabadi et al., 2020).

As mentioned above, the ability of bioSeNPs was investigated for antifungal activity against *Candida albicans*. All experiments were performed in triplicate. After 24 h incubation, the absorbance of each well was measured by a microtitre plate reader at 600 nm. Also, all strains were found to be resistant to selenite concentration equivalent to the MIC of the nanoparticles.

### Effect of bioSeNPs on biofilm formation

The inhibitory effect of bioSeNPs on formation of biofilm by different bacteria was evaluated using 96‐well plates. In this experiment, 10^8^ CFU/mL of bacterial strains, including, *A. baumannii*, *P. aeruginosa*, *K. pneumonia* and *S. aureus*, were prepared in TSB containing 2% glucose and 0.0, 0.25, 0.5, 1.0 and 1.5 mg of bioSeNPs. After adding to each well, the plate was incubated for 48 h at 37°C without shaking. The cultured wells were washed twice with physiological saline solution, and 100 μL methanol was added to each well. After 20 min, 200 μL crystal violet solution (1% w/v) was added. After washing by physiological saline solution, 150 μL glacial acetic acid (33% v/v) was used to solubilize the crystal violet dye bound to biofilm. Finally, absorbance at 590 nm was obtained by a microtitre plate reader. The same culture condition without nanoparticles was used as a control (Allkja et al., 2020; Balouiri et al., 2016; Shakibaie et al., 2015).

### Biogenic selenium nanoparticle cytotoxicity

Among 40 different hepatic cell lines, the HepG2 cell line has gained attention due to its easy handling, various morphological features of liver parenchymal cells, wide range of applications in scientific research and several enzymes for biotransformation (Rudzok et al., 2010; Senthilraja & Kathiresan, 2015). According to Arzumanian et al., 2021, HepG2 cell line was used in 76% of manuscripts (between 44,971 articles) using human hepatic cell lines by searching this term on PubMed (accessed on 23 November 2021).

HUVEC and Hep‐G2 cell lines were prepared from the Institute of Biochemistry and Biophysics and Virology and Molecular Laboratory of the University of Tehran, respectively. These cell lines were cultured on DMEM (containing L‐Glutamine, 2%; penicillin/streptomycin, 1%; and FBS, 10%). After the incubation in 5% CO_2_–95% air incubator at 37°C for 24 h, HUVEC, and Hep‐G2 cells were collected using trypsin and inoculated in 96‐well plates (10^4^ cells/mL), and incubated overnight at 37°C. A range of 0.5–4 mg/mL of bioSeNPs was prepared in the DMEM medium and added to each well, and incubation was done as above. Then, culture media were removed, and an MTT solution was added to each well. The plate was incubated at 37°C for 4 h. By removing other MTT solution, DMSO was added, and absorbance was evaluated at 570 nm.

Cell viability was calculated by the formula:

$$\text{cell viability}\;\left(\%\right)=\frac{A₁-A₂}{C-A₂}\times 100$$

When *A*_1_, *A*_2_ and *C* are OD_treatment_, OD_blank_ and OD_control_, respectively (Mohammadi‐Yeganeh et al., 2016).

### Antioxidant effect of selenium nanoparticles

The antioxidant activity of bioSeNPs was measured by evaluating DPPH (1,1‐diphenyl‐2‐picrylhydrazyl) colour change from purple to yellow. When a nanoparticle has antioxidant activity, it can donate an electron to DPPH and reduce it. In this experiment, a range of 20–640 μg/mL of SeNPs was used to assay antioxidant activity. In this regard, 100 μL of DPPH solution (0.2 mM prepared in methanol) was added to 100 μL of different concentration of bioSeNPs, and 96‐well plate was incubated at room temperature for 30 min in the darkness. A negative and positive controls were prepared by replacing the bioSeNPs with distilled water, and ascorbic acid was applied as a positive control. Absorbance was measured by Eliza reader at 517 nm. The antioxidant activity was obtained by using the following formula:

$$\text{DPPH radical scavenging ability}=\left[1-\frac{A₁-A₂}{A₃}\right]\times 100$$

where *A*_1_ is sample absorbance with DPPH solution, *A*_2_ is the absorbance of the bioSeNPs containing wells without DPPH solution, and *A*_3_ is the absorbance of the control solution (Eid et al., 2023; Forootanfar et al., 2014).

### Data statistical analysis

All experiments were performed in three replicates. Analysis of variance (ANOVA) and Tukey's comparison test (with a confidence level of 95% (*p* < 0.05)) were applied for statistical analyses by using SPSS (version 22).

## AUTHOR CONTRIBUTIONS

**Elham Lashani:** Conceptualization; data curation; formal analysis; investigation; methodology; software; visualization; writing – original draft; writing – review and editing. **Hamid Moghimi:** Conceptualization; project administration; resources; supervision; validation; writing – original draft; writing – review and editing. **Raymond J. Turner:** Conceptualization; project administration; resources; supervision; validation; writing – original draft; writing – review and editing. **Mohammad Ali Amoozegar:** Conceptualization; project administration; resources; supervision; validation; writing – original draft; writing – review and editing.

## CONFLICT OF INTEREST STATEMENT

The authors declare that they have no competing interests.

## Data Availability

All data are included in the manuscript and additional information, and further queries about sharing data can be directed to the corresponding author.

## References

[mbt270013-bib-0001] Abu‐Elghait, M. , Hasanin, M. , Hashem, A.H. & Salem, S.S. (2021) Ecofriendly novel synthesis of tertiary composite based on cellulose and myco‐synthesized selenium nanoparticles: characterization, antibiofilm and biocompatibility. International Journal of Biological Macromolecules, 175, 294–303. 33571585 10.1016/j.ijbiomac.2021.02.040

[mbt270013-bib-0002] Afzal, B. , Yasin, D. , Naaz, H. , Sami, N. , Zaki, A. , Rizvi, M.A. et al. (2021) Biomedical potential of *Anabaena variabilis* NCCU‐441 based selenium nanoparticles and their comparison with commercial nanoparticles. Scientific Reports, 11, 13507. 34188065 10.1038/s41598-021-91738-7 PMC8242014

[mbt270013-bib-0003] Ahluwalia, S. , Prakash, N.T. , Prakash, R. & Pal, B. (2016) Improved degradation of methyl orange dye using bio‐co‐catalyst Se nanoparticles impregnated ZnS photocatalyst under UV irradiation. Chemical Engineering Journal, 306, 1041–1048.

[mbt270013-bib-0004] Ahmad, A. , Ullah, S. , Khan, A. , Ahmad, W. , Khan, A.U. , Khan, U.A. et al. (2020) Graphene oxide selenium nanorod composite as a stable electrode material for energy storage devices. Applied Nanoscience, 10, 1243–1255.

[mbt270013-bib-0005] Ahmad, M.S. , Yasser, M.M. , Sholkamy, E.N. , Ali, A.M. & Mehanni, M.M. (2015) Anticancer activity of biostabilized selenium nanorods synthesized by *Streptomyces bikiniensis* strain Ess_amA‐1. International Journal of Nanomedicine, 10, 3389–3401. 26005349 10.2147/IJN.S82707 PMC4428361

[mbt270013-bib-0006] Akçay, F.A. & Avcı, A. (2020) Effects of process conditions and yeast extract on the synthesis of selenium nanoparticles by a novel indigenous isolate *Bacillus* sp. EKT1 and characterization of nanoparticles. Archives of Microbiology, 202, 2233–2243. 32533206 10.1007/s00203-020-01942-8

[mbt270013-bib-0007] Allkja, J. , Bjarnsholt, T. , Coenye, T. , Cos, P. , Fallarero, A. , Harrison, J.J. et al. (2020) Minimum information guideline for spectrophotometric and fluorometric methods to assess biofilm formation in microplates. Biofilms, 2, 100010. 10.1016/j.bioflm.2019.100010 PMC7798448 33447797

[mbt270013-bib-0008] Al‐Quraishy, S. , Dkhil, M.A. & Abdel Moneim, A.E. (2015) Anti‐hyperglycemic activity of selenium nanoparticles in streptozotocin‐induced diabetic rats. International Journal of Nanomedicine, 10, 6741–6756. 26604749 10.2147/IJN.S91377 PMC4631434

[mbt270013-bib-0009] Arora, A. , Lashani, E. & Turner, R.J. (2024) Bacterial synthesis of metal nanoparticles as antimicrobials. Microbial Biotechnology, 17, e14549. Available from: 10.1111/1751-7915.14549 39150434 PMC11328525

[mbt270013-bib-0010] Arzumanian, V.A. , Kiseleva, O.I. & Poverennaya, E.V. (2021) The curious case of the HepG2 cell line: 40 years of expertise. International Journal of Molecular Sciences, 22, 13135. 34884942 10.3390/ijms222313135 PMC8658661

[mbt270013-bib-0011] Ashengroph, M. & Hosseini, S.‐R. (2021) A newly isolated *Bacillus amyloliquefaciens* SRB04 for the synthesis of selenium nanoparticles with potential antibacterial properties. International Microbiology, 24, 103–114. 33124680 10.1007/s10123-020-00147-9

[mbt270013-bib-0012] Ashengroph, M. & Tozandehjani, S. (2022) Optimized resting cell method for green synthesis of selenium nanoparticles from a new *Rhodotorula mucilaginosa* strain. Process Biochemistry, 116, 197–205.

[mbt270013-bib-0013] Baggio, G. , Groves, R.A. , Chignola, R. , Piacenza, E. , Presentato, A. , Lewis, I.A. et al. (2021) Untargeted metabolomics investigation on selenite reduction to elemental selenium by *Bacillus mycoides* SeITE01. Frontiers in Microbiology, 12, 711000. 34603239 10.3389/fmicb.2021.711000 PMC8481872

[mbt270013-bib-0014] Bakhtiari‐Sardari, A. , Mashreghi, M. , Eshghi, H. , Behnam‐Rasouli, F. , Lashani, E. & Shahnavaz, B. (2020) Comparative evaluation of silver nanoparticles biosynthesis by two cold‐tolerant Streptomyces strains and their biological activities. Biotechnology Letters, 42, 1985–1999. 32462288 10.1007/s10529-020-02921-1

[mbt270013-bib-0015] Balouiri, M. , Sadiki, M. & Ibnsouda, S.K. (2016) Methods for in vitro evaluating antimicrobial activity: a review. Journal of Pharmaceutical Analysis, 6, 71–79. 29403965 10.1016/j.jpha.2015.11.005 PMC5762448

[mbt270013-bib-0016] Baran, M.F. (2019) Synthesis and antimicrobial applications of silver nanoparticles from *artemisia absinthium plant* . Journal of Biological and Chemical Research, 6, 96–103.

[mbt270013-bib-0017] Baran, M.F. , Keskin, C. , Baran, A. , Kurt, K. , İpek, P. , Eftekhari, A. et al. (2023) Green synthesis and characterization of selenium nanoparticles (Se NPs) from the skin (testa) of *Pistacia vera* L.(*Siirt pistachio*) and investigation of antimicrobial and anticancer potentials. Biomass Conversion and Biorefinery, 1–11. Available from: 10.1007/s13399-023-04366-8

[mbt270013-bib-0018] Barani, M. , Masoudi, M. , Mashreghi, M. , Makhdoumi, A. & Eshghi, H. (2021) Cell‐free extract assisted synthesis of ZnO nanoparticles using aquatic bacterial strains: biological activities and toxicological evaluation. International Journal of Pharmaceutics, 606, 120878. 34265392 10.1016/j.ijpharm.2021.120878

[mbt270013-bib-0019] Beheshti, N. , Soflaei, S. , Shakibaie, M. , Yazdi, M.H. , Ghaffarifar, F. , Dalimi, A. et al. (2013) Efficacy of biogenic selenium nanoparticles against Leishmania major: in vitro and in vivo studies. Journal of Trace Elements in Medicine and Biology, 27, 203–207. 23219368 10.1016/j.jtemb.2012.11.002

[mbt270013-bib-0020] Bharathi, S. , Kumaran, S. , Suresh, G. , Ramesh, M. , Thangamani, V. , Pugazhvendan, S. et al. (2020) Extracellular synthesis of nanoselenium from fresh water bacteria *Bacillus* sp., and its validation of antibacterial and cytotoxic potential. Biocatalysis and Agricultural Biotechnology, 27, 101655.

[mbt270013-bib-0021] Bisht, N. , Phalswal, P. & Khanna, P.K. (2022) Selenium nanoparticles: a review on synthesis and biomedical applications. Materials Advances, 3, 1415–1431.

[mbt270013-bib-0022] Borghese, R. , Baccolini, C. , Francia, F. , Sabatino, P. , Turner, R.J. & Zannoni, D. (2014) Reduction of chalcogen oxyanions and generation of nanoprecipitates by the photosynthetic bacterium *Rhodobacter capsulatus* . Journal of Hazardous Materials, 269, 24–30. 24462199 10.1016/j.jhazmat.2013.12.028

[mbt270013-bib-0023] Bulgarini, A. , Lampis, S. , Turner, R.J. & Vallini, G. (2021) Biomolecular composition of capping layer and stability of biogenic selenium nanoparticles synthesized by five bacterial species. Microbial Biotechnology, 14, 198–212. 33068075 10.1111/1751-7915.13666 PMC7888468

[mbt270013-bib-0024] Cremonini, E. , Boaretti, M. , Vandecandelaere, I. , Zonaro, E. , Coenye, T. , Lleo, M.M. et al. (2018) Biogenic selenium nanoparticles synthesized by *Stenotrophomonas maltophilia* Se ITE 02 loose antibacterial and antibiofilm efficacy as a result of the progressive alteration of their organic coating layer. Microbial Biotechnology, 11, 1037–1047. 29635772 10.1111/1751-7915.13260 PMC6196382

[mbt270013-bib-0025] Cremonini, E. , Zonaro, E. , Donini, M. , Lampis, S. , Boaretti, M. , Dusi, S. et al. (2016) Biogenic selenium nanoparticles: characterization, antimicrobial activity and effects on human dendritic cells and fibroblasts. Microbial Biotechnology, 9, 758–771. 27319803 10.1111/1751-7915.12374 PMC5072192

[mbt270013-bib-0026] Dumore, N.S. & Mukhopadhyay, M. (2020) Antioxidant properties of aqueous selenium nanoparticles (ASeNPs) and its catalysts activity for 1, 1‐diphenyl‐2‐picrylhydrazyl (DPPH) reduction. Journal of Molecular Structure, 1205, 127637.

[mbt270013-bib-0027] Eid, N. , Yosri, N. , El‐Seedi, H.R. , Awad, H.M. & Emam, H.E. (2023) Ag@ Sidr honey nanocomposite: chemical profiles, antioxidant and microbicide procurator. Biocatalysis and Agricultural Biotechnology, 51, 102788.

[mbt270013-bib-0028] El‐Fakharany, E.M. , Abu‐Serie, M.M. , Ibrahim, A. & Eltarahony, M. (2023) Anticancer activity of lactoferrin‐coated biosynthesized selenium nanoparticles for combating different human cancer cells via mediating apoptotic effects. Scientific Reports, 13, 9579. 37311791 10.1038/s41598-023-36492-8 PMC10264462

[mbt270013-bib-0029] El‐Saadony, M.T. , Saad, A.M. , Taha, T.F. , Najjar, A.A. , Zabermawi, N.M. , Nader, M.M. et al. (2021) Selenium nanoparticles from *lactobacillus paracasei* HM1 capable of antagonizing animal pathogenic fungi as a new source from human breast milk. Saudi Journal of Biological Sciences, 28, 6782–6794. 34866977 10.1016/j.sjbs.2021.07.059 PMC8626219

[mbt270013-bib-0030] Elshaer, S.L. & Shaaban, M.I. (2021) Inhibition of quorum sensing and virulence factors of *Pseudomonas aeruginosa* by biologically synthesized gold and selenium nanoparticles. Antibiotics, 10, 1461. 34943673 10.3390/antibiotics10121461 PMC8698379

[mbt270013-bib-0031] Eramabadi, P. , Masoudi, M. , Makhdoumi, A. & Mashreghi, M. (2020) Microbial cell lysate supernatant (CLS) alteration impact on platinum nanoparticles fabrication, characterization, antioxidant and antibacterial activity. Materials Science and Engineering: C, 117, 111292. 32919653 10.1016/j.msec.2020.111292

[mbt270013-bib-0032] Faramarzi, S. , Anzabi, Y. & Jafarizadeh‐Malmiri, H. (2020) Nanobiotechnology approach in intracellular selenium nanoparticle synthesis using *Saccharomyces cerevisiae*—fabrication and characterization. Archives of Microbiology, 202, 1203–1209. 32077990 10.1007/s00203-020-01831-0

[mbt270013-bib-0033] Fernández‐Llamosas, H. , Castro, L. , Blázquez, M.L. , Díaz, E. & Carmona, M. (2016) Biosynthesis of selenium nanoparticles by *Azoarcus* sp. CIB. Microbial Cell Factories, 15, 1–10. 27301452 10.1186/s12934-016-0510-y PMC4908764

[mbt270013-bib-0034] Filipović, N. , Ušjak, D. , Milenković, M.T. , Zheng, K. , Liverani, L. , Boccaccini, A.R. et al. (2021) Comparative study of the antimicrobial activity of selenium nanoparticles with different surface chemistry and structure. Frontiers in Bioengineering and Biotechnology, 8, 624621. 33569376 10.3389/fbioe.2020.624621 PMC7869925

[mbt270013-bib-0035] Forootanfar, H. , Adeli‐Sardou, M. , Nikkhoo, M. , Mehrabani, M. , Amir‐Heidari, B. , Shahverdi, A.R. et al. (2014) Antioxidant and cytotoxic effect of biologically synthesized selenium nanoparticles in comparison to selenium dioxide. Journal of Trace Elements in Medicine and Biology, 28, 75–79. 24074651 10.1016/j.jtemb.2013.07.005

[mbt270013-bib-0036] Gold, K. , Slay, B. , Knackstedt, M. & Gaharwar, A.K. (2018) Antimicrobial activity of metal and metal‐oxide based nanoparticles. Advances in Therapy, 1, 1700033.

[mbt270013-bib-0037] Guo, M. , Li, Y. , Lin, Z. , Zhao, M. , Xiao, M. , Wang, C. et al. (2017) Surface decoration of selenium nanoparticles with curcumin induced HepG2 cell apoptosis through ROS mediated p53 and AKT signaling pathways. RSC Advances, 7, 52456–52464.

[mbt270013-bib-0038] Hamza, F. , Vaidya, A. , Apte, M. , Kumar, A.R. & Zinjarde, S. (2017) Selenium nanoparticle‐enriched biomass of *Yarrowia lipolytica* enhances growth and survival of Artemia salina. Enzyme and Microbial Technology, 106, 48–54. 28859809 10.1016/j.enzmictec.2017.07.002

[mbt270013-bib-0039] Hashem, A.H. , Khalil, A.M.A. , Reyad, A.M. & Salem, S.S. (2021) Biomedical applications of mycosynthesized selenium nanoparticles using *Penicillium expansum* ATTC 36200. Biological Trace Element Research, 119, 1–11. 10.1007/s12011-020-02506-z 33387272

[mbt270013-bib-0040] Hassanshahian, M. , Tebyanian, H. & Cappello, S. (2012) Isolation and characterization of two crude oil‐degrading yeast strains, *Yarrowia lipolytica* PG‐20 and PG‐32, from the Persian Gulf. Marine Pollution Bulletin, 64, 1386–1391. 22622152 10.1016/j.marpolbul.2012.04.020

[mbt270013-bib-0041] Hosseini, F. , Hadian, M. , Lashani, E. & Moghimi, H. (2023) Simultaneous bioreduction of tellurite and selenite by *Yarrowia lipolytica*, *Trichosporon cutaneum*, and their co‐culture along with characterization of biosynthesized Te–Se nanoparticles. Microbial Cell Factories, 22, 193. 37749532 10.1186/s12934-023-02204-0 PMC10519092

[mbt270013-bib-0042] Hosseini, F. , Lashani, E. & Moghimi, H. (2023) Simultaneous bioremediation of phenol and tellurite by *Lysinibacillus* sp. EBL303 and characterization of biosynthesized Te nanoparticles. Scientific Reports, 13, 1243. 36690691 10.1038/s41598-023-28468-5 PMC9870877

[mbt270013-bib-0043] Huang, X. , Chen, X. , Chen, Q. , Yu, Q. , Sun, D. & Liu, J. (2016) Investigation of functional selenium nanoparticles as potent antimicrobial agents against superbugs. Acta Biomaterialia, 30, 397–407. 26518106 10.1016/j.actbio.2015.10.041

[mbt270013-bib-0044] Jach, M.E. & Malm, A. (2022) *Yarrowia lipolytica* as an alternative and valuable source of nutritional and bioactive compounds for humans. Molecules, 27, 2300. 35408699 10.3390/molecules27072300 PMC9000428

[mbt270013-bib-0045] Jach, M.E. , Sajnaga, E. , Janeczko, M. , Juda, M. , Kochanowicz, E. , Baj, T. et al. (2021) Production of enriched in B vitamins biomass of *Yarrowia lipolytica* grown in biofuel waste. Saudi Journal of Biological Sciences, 28, 2925–2932. 34025170 10.1016/j.sjbs.2021.02.027 PMC8117029

[mbt270013-bib-0046] Jain, R. , Jordan, N. , Weiss, S. , Foerstendorf, H. , Heim, K. , Kacker, R. et al. (2015) Extracellular polymeric substances govern the surface charge of biogenic elemental selenium nanoparticles. Environmental Science & Technology, 49, 1713–1720. 25536371 10.1021/es5043063

[mbt270013-bib-0047] Jamkhande, P.G. , Ghule, N.W. , Bamer, A.H. & Kalaskar, M.G. (2019) Metal nanoparticles synthesis: an overview on methods of preparation, advantages and disadvantages, and applications. Journal of Drug Delivery Science and Technology, 53, 101174.

[mbt270013-bib-0048] Khiralla, G.M. & El‐Deeb, B.A. (2015) Antimicrobial and antibiofilm effects of selenium nanoparticles on some foodborne pathogens. LWT‐ Food Science and Technology, 63, 1001–1007.

[mbt270013-bib-0049] Kieliszek, M. , Błażejak, S. , Gientka, I. & Bzducha‐Wróbel, A. (2015) Accumulation and metabolism of selenium by yeast cells. Applied Microbiology and Biotechnology, 99, 5373–5382. 26003453 10.1007/s00253-015-6650-x PMC4464373

[mbt270013-bib-0050] Klabunde, K.J. (1994) Free atoms, clusters, and nanoscale particles. Cambridge, MA: Academic Press.

[mbt270013-bib-0051] Kora, A.J. (2018) *Bacillus cereus*, selenite‐reducing bacterium from contaminated lake of an industrial area: a renewable nanofactory for the synthesis of selenium nanoparticles. Bioresources and Bioprocessing, 5, 1–12.

[mbt270013-bib-0052] Kora, A.J. & Rastogi, L. (2016) Biomimetic synthesis of selenium nanoparticles by *Pseudomonas aeruginosa* ATCC 27853: an approach for conversion of selenite. Journal of Environmental Management, 181, 231–236. 27353373 10.1016/j.jenvman.2016.06.029

[mbt270013-bib-0053] Kumar, A. , Prasad, B. , Manjhi, J. & Prasad, K.S. (2020) Antioxidant activity of selenium nanoparticles biosynthesized using a cell‐free extract of *Geobacillus* . Toxicological and Environmental Chemistry, 102, 556–567.

[mbt270013-bib-0054] Lampis, S. , Zonaro, E. , Bertolini, C. , Bernardi, P. , Butler, C.S. & Vallini, G. (2014) Delayed formation of zero‐valent selenium nanoparticles by *Bacillus mycoides* SeITE01 as a consequence of selenite reduction under aerobic conditions. Microbial Cell Factories, 13, 1–14. 24606965 10.1186/1475-2859-13-35 PMC3975340

[mbt270013-bib-0055] Lampis, S. , Zonaro, E. , Bertolini, C. , Cecconi, D. , Monti, F. , Micaroni, M. et al. (2017) Selenite biotransformation and detoxification by *Stenotrophomonas maltophilia* SeITE02: novel clues on the route to bacterial biogenesis of selenium nanoparticles. Journal of Hazardous Materials, 324, 3–14. 26952084 10.1016/j.jhazmat.2016.02.035

[mbt270013-bib-0056] Lashani, E. , Moghimi, H. , Turner, R.J. & Amoozegar, M.A. (2023) Selenite bioreduction by a consortium of halophilic/halotolerant bacteria and/or yeasts in saline media. Environmental Pollution, 331, 121948. 37270053 10.1016/j.envpol.2023.121948

[mbt270013-bib-0057] Lazard, M. , Blanquet, S. , Fisicaro, P. , Labarraque, G. & Plateau, P. (2010) Uptake of selenite by *Saccharomyces cerevisiae* involves the high and low affinity orthophosphate transporters. The Journal of Biological Chemistry, 285, 32029–32037. 20688911 10.1074/jbc.M110.139865 PMC2952204

[mbt270013-bib-1000] Lian, S. , Diko, C.S. , Yan, Y. , Li, Z. , Zhang, H. , Ma, Q. & Qu, Y. (2019) Characterization of biogenic selenium nanoparticles derived from cell‐free extracts of a novel yeast Magnusiomyces ingens. 3 Biotech, 9, 1–8. 10.1007/s13205-019-1748-y PMC6527717 31114745

[mbt270013-bib-0058] Long, Q. , Cui, L.‐k. , He, S.‐b. , Sun, J. , Chen, Q.‐z. , Bao, H.‐d. et al. (2023) Preparation, characteristics and cytotoxicity of green synthesized selenium nanoparticles using *Paenibacillus motobuensis* LY5201 isolated from the local specialty food of longevity area. Scientific Reports, 13, 53. 36593245 10.1038/s41598-022-26396-4 PMC9807572

[mbt270013-bib-0059] Malhotra, S. , Welling, M. , Mantri, S. & Desai, K. (2016) In vitro and in vivo antioxidant, cytotoxic, and anti‐chronic inflammatory arthritic effect of selenium nanoparticles. Journal of Biomedical Materials Research. Part B, Applied Biomaterials, 104, 993–1003. 25994972 10.1002/jbm.b.33448

[mbt270013-bib-0060] McDermott, J.R. , Rosen, B.P. & Liu, Z. (2010) Jen1p: a high affinity selenite transporter in yeast. Molecular Biology of the Cell, 21, 3934–3941. 20861301 10.1091/mbc.E10-06-0513 PMC2982120

[mbt270013-bib-0061] Menon, S. , Agarwal, H. , Kumar, S.V. & Rajeshkumar, S. (2019) Biomemetic synthesis of selenium nanoparticles and its biomedical applications. In: Green synthesis, characterization and applications of nanoparticles. Amsterdam, Netherlands: Elsevier, pp. 165–197.

[mbt270013-bib-0062] Mohammadi‐Yeganeh, S. , Paryan, M. , Arefian, E. , Vasei, M. , Ghanbarian, H. , Mahdian, R. et al. (2016) MicroRNA‐340 inhibits the migration, invasion, and metastasis of breast cancer cells by targeting Wnt pathway. Tumor Biology, 37, 8993–9000. 26758430 10.1007/s13277-015-4513-9

[mbt270013-bib-0063] Panahi‐Kalamuei, M. , Salavati‐Niasari, M. & Hosseinpour‐Mashkani, S.M. (2014) Facile microwave synthesis, characterization, and solar cell application of selenium nanoparticles. Journal of Alloys and Compounds, 617, 627–632.

[mbt270013-bib-0064] Piacenza, E. , Presentato, A. , Ambrosi, E. , Speghini, A. , Turner, R.J. , Vallini, G. et al. (2018) Physical–chemical properties of biogenic selenium nanostructures produced by *Stenotrophomonas maltophilia* SeITE02 and *Ochrobactrum* sp. MPV1. Frontiers in Microbiology, 9, 3178. 30619230 10.3389/fmicb.2018.03178 PMC6306038

[mbt270013-bib-0065] Piacenza, E. , Presentato, A. , Zonaro, E. , Lemire, J.A. , Demeter, M. , Vallini, G. et al. (2017) Antimicrobial activity of biogenically produced spherical Se‐nanomaterials embedded in organic material against *Pseudomonas aeruginosa* and *Staphylococcus aureus* strains on hydroxyapatite‐coated surfaces. Microbial Biotechnology, 10, 804–818. 28233476 10.1111/1751-7915.12700 PMC5481514

[mbt270013-bib-0066] Piacenza, E. , Sule, K. , Presentato, A. , Wells, F. , Turner, R.J. & Prenner, E.J. (2023) Impact of biogenic and Chemogenic selenium nanoparticles on model eukaryotic lipid membranes. Langmuir, 39, 10406–10419. 37462214 10.1021/acs.langmuir.3c00718 PMC10399287

[mbt270013-bib-0067] Prasad, K.S. , Vaghasiya, J.V. , Soni, S.S. , Patel, J. , Patel, R. , Kumari, M. et al. (2015) Microbial selenium nanoparticles (SeNPs) and their application as a sensitive hydrogen peroxide biosensor. Applied Biochemistry and Biotechnology, 177, 1386–1393. 26319569 10.1007/s12010-015-1814-9

[mbt270013-bib-0068] Presentato, A. , Piacenza, E. , Anikovskiy, M. , Cappelletti, M. , Zannoni, D. & Turner, R.J. (2016) *Rhodococcus aetherivorans* BCP1 as cell factory for the production of intracellular tellurium nanorods under aerobic conditions. Microbial Cell Factories, 15, 1–14. 27978836 10.1186/s12934-016-0602-8 PMC5157098

[mbt270013-bib-0069] Presentato, A. , Piacenza, E. , Anikovskiy, M. , Cappelletti, M. , Zannoni, D. & Turner, R.J. (2018) Biosynthesis of selenium‐nanoparticles and‐nanorods as a product of selenite bioconversion by the aerobic bacterium *Rhodococcus aetherivorans* BCP1. New Biotechnology, 41, 1–8. 29174512 10.1016/j.nbt.2017.11.002

[mbt270013-bib-0070] Rajagopal, G. , Nivetha, A. , Ilango, S. , Muthudevi, G.P. , Prabha, I. & Arthimanju, R. (2021) Phytofabrication of selenium nanoparticles using *Azolla pinnata*: evaluation of catalytic properties in oxidation, antioxidant and antimicrobial activities. Journal of Environmental Chemical Engineering, 9, 105483.

[mbt270013-bib-0071] Ramya, S. , Shanmugasundaram, T. & Balagurunathan, R. (2015) Biomedical potential of actinobacterially synthesized selenium nanoparticles with special reference to anti‐biofilm, anti‐oxidant, wound healing, cytotoxic and anti‐viral activities. Journal of Trace Elements in Medicine and Biology, 32, 30–39. 26302909 10.1016/j.jtemb.2015.05.005

[mbt270013-bib-0072] Ramya, S. , Shanmugasundaram, T. & Balagurunathan, R. (2020) Actinobacterial enzyme mediated synthesis of selenium nanoparticles for antibacterial, mosquito larvicidal and anthelminthic applications. Particulate Science and Technology, 38, 63–72.

[mbt270013-bib-0073] Rudzok, S. , Schlink, U. , Herbarth, O. & Bauer, M. (2010) Measuring and modeling of binary mixture effects of pharmaceuticals and nickel on cell viability/cytotoxicity in the human hepatoma derived cell line HepG2. Toxicology and Applied Pharmacology, 244, 336–343. 20132835 10.1016/j.taap.2010.01.012

[mbt270013-bib-0074] Ruocco, M.H. , Chan, C.S. , Hanson, T.E. & Church, T.M. (2014) Characterization and distribution of selenite reduction products in cultures of the marine yeast *Rhodotorula mucilaginosa*‐13B. Geomicrobiology Journal, 31, 769–778.

[mbt270013-bib-0075] Salem, S.S. (2022) Bio‐fabrication of selenium nanoparticles using Baker's yeast extract and its antimicrobial efficacy on food borne pathogens. Applied Biochemistry and Biotechnology, 194, 1898–1910. 34994951 10.1007/s12010-022-03809-8

[mbt270013-bib-0076] Samant, S. , Naik, M. , Parulekar, K. , Charya, L. & Vaigankar, D. (2018) Selenium reducing *Citrobacter fruendii* strain KP6 from Mandovi estuary and its potential application in selenium nanoparticle synthesis. Proceedings of the National Academy of Sciences, India Section B, 88, 747–754.

[mbt270013-bib-0077] San Keskin, N.O. , Akbal Vural, O. & Abaci, S. (2020) Biosynthesis of noble selenium nanoparticles from *Lysinibacillus* sp. NOSK for Antimicrobial, Antibiofilm Activity, and Biocompatibility. Geomicrobiology Journal, 37, 919–928.

[mbt270013-bib-0078] Senthilraja, P. & Kathiresan, K. (2015) In vitro cytotoxicity MTT assay in Vero, HepG2 and MCF‐7 cell lines study of marine yeast. Journal of Applied Pharmaceutical Science, 5, 80–84.

[mbt270013-bib-0079] Shakibaie, M. , Forootanfar, H. , Golkari, Y. , Mohammadi‐Khorsand, T. & Shakibaie, M.R. (2015) Anti‐biofilm activity of biogenic selenium nanoparticles and selenium dioxide against clinical isolates of *Staphylococcus aureus*, *Pseudomonas aeruginosa*, and *Proteus mirabilis* . Journal of Trace Elements in Medicine and Biology, 29, 235–241. 25175509 10.1016/j.jtemb.2014.07.020

[mbt270013-bib-0080] Shoeibi, S. & Mashreghi, M. (2017) Biosynthesis of selenium nanoparticles using *enterococcus faecalis* and evaluation of their antibacterial activities. Journal of Trace Elements in Medicine and Biology, 39, 135–139. 27908405 10.1016/j.jtemb.2016.09.003

[mbt270013-bib-0081] Sonkusre, P. & Cameotra, S.S. (2015) Biogenic selenium nanoparticles inhibit *Staphylococcus aureus* adherence on different surfaces. Colloids and Surfaces. B, Biointerfaces, 136, 1051–1057. 26590898 10.1016/j.colsurfb.2015.10.052

[mbt270013-bib-0082] Spyridopoulou, K. , Tryfonopoulou, E. , Aindelis, G. , Ypsilantis, P. , Sarafidis, C. , Kalogirou, O. et al. (2021) Biogenic selenium nanoparticles produced by *lactobacillus casei* ATCC 393 inhibit colon cancer cell growth in vitro and in vivo. Nanoscale Advances, 3, 2516–2528. 36134160 10.1039/d0na00984a PMC9417964

[mbt270013-bib-0083] Srivastava, N. & Mukhopadhyay, M. (2015) Green synthesis and structural characterization of selenium nanoparticles and assessment of their antimicrobial property. Bioprocess and Biosystems Engineering, 38, 1723–1730. 25972036 10.1007/s00449-015-1413-8

[mbt270013-bib-0084] Srivastava, P. & Kowshik, M. (2016) Anti‐neoplastic selenium nanoparticles from *Idiomarina* sp. PR58‐8. Enzyme and Microbial Technology, 95, 192–200. 27866615 10.1016/j.enzmictec.2016.08.002

[mbt270013-bib-0085] Tugarova, A.V. , Mamchenkova, P.V. , Dyatlova, Y.A. & Kamnev, A.A. (2018) FTIR and Raman spectroscopic studies of selenium nanoparticles synthesised by the bacterium *Azospirillum thiophilum* . Spectrochimica Acta Part A: Molecular Spectroscopy, 192, 458–463. 10.1016/j.saa.2017.11.050 29220816

[mbt270013-bib-0086] Wadhwani, S.A. , Gorain, M. , Banerjee, P. , Shedbalkar, U.U. , Singh, R. , Kundu, G.C. et al. (2017) Green synthesis of selenium nanoparticles using *Acinetobacter* sp. SW30: optimization, characterization and its anticancer activity in breast cancer cells. International Journal of Nanomedicine, 12, 6841–6855. 28979122 10.2147/IJN.S139212 PMC5602452

[mbt270013-bib-0087] Wang, X. , Wang, S. , Pan, X. & Gadd, G.M. (2019) Heteroaggregation of soil particulate organic matter and biogenic selenium nanoparticles for remediation of elemental mercury contamination. Chemosphere, 221, 486–492. 30654263 10.1016/j.chemosphere.2019.01.073

[mbt270013-bib-0088] Wang, Y. , Ye, Q. , Sun, Y. , Jiang, Y. , Meng, B. , Du, J. et al. (2022) Selenite reduction by *Proteus* sp. YS02: new insights revealed by comparative transcriptomics and antibacterial effectiveness of the biogenic Se0 nanoparticles. Frontiers in Microbiology, 13, 845321. 35359742 10.3389/fmicb.2022.845321 PMC8960269

[mbt270013-bib-0089] Xu, C. , Qiao, L. , Guo, Y. , Ma, L. & Cheng, Y. (2018) Preparation, characteristics and antioxidant activity of polysaccharides and proteins‐capped selenium nanoparticles synthesized by *lactobacillus casei* ATCC 393. Carbohydrate Polymers, 195, 576–585. 29805014 10.1016/j.carbpol.2018.04.110

[mbt270013-bib-0090] Yang, F. , Tang, Q. , Zhong, X. , Bai, Y. , Chen, T. , Zhang, Y. et al. (2012) Surface decoration by spirulina polysaccharide enhances the cellular uptake and anticancer efficacy of selenium nanoparticles. International Journal of Nanomedicine, 7, 835–844. 22359460 10.2147/IJN.S28278 PMC3284226

[mbt270013-bib-0091] Yang, R. , Chen, Z. , Hu, P. , Zhang, S. & Luo, G. (2022) Two‐stage fermentation enhanced single‐cell protein production by *Yarrowia lipolytica* from food waste. Bioresource Technology, 361, 127677. 35878768 10.1016/j.biortech.2022.127677

[mbt270013-bib-0092] Yildirim, A. , Acay, H. & Baran, F. (2022) Synthesis and characterisation of mushroom‐based nanocomposite and its efficiency on dye biosorption via antimicrobial activity. International Journal of Environmental Analytical Chemistry, 102, 1545–1562.

[mbt270013-bib-0093] Yosri, N. , Khalifa, S.A. , Guo, Z. , Xu, B. , Zou, X. & El‐Seedi, H.R. (2021) Marine organisms: Pioneer natural sources of polysaccharides/proteins for green synthesis of nanoparticles and their potential applications. International Journal of Biological Macromolecules, 193, 1767–1798. 34752793 10.1016/j.ijbiomac.2021.10.229

[mbt270013-bib-0094] Zhang, H. , Li, Z. , Dai, C. , Wang, P. , Fan, S. , Yu, B. et al. (2021) Antibacterial properties and mechanism of selenium nanoparticles synthesized by *Providencia* sp. DCX. Environmental Research, 194, 110630. 33345899 10.1016/j.envres.2020.110630

[mbt270013-bib-0095] Zhang, H. , Zhou, H. , Bai, J. , Li, Y. , Yang, J. , Ma, Q. et al. (2019) Biosynthesis of selenium nanoparticles mediated by fungus *Mariannaea* sp. HJ and their characterization. Colloids and Surfaces A‐Physicochemical and Engineering Aspects, 571, 9–16.

[mbt270013-bib-0096] Zonaro, E. , Lampis, S. , Turner, R.J. , Qazi, S.J.S. & Vallini, G. (2015) Biogenic selenium and tellurium nanoparticles synthesized by environmental microbial isolates efficaciously inhibit bacterial planktonic cultures and biofilms. Frontiers in Microbiology, 6, 584. 26136728 10.3389/fmicb.2015.00584 PMC4468835

